# Efferocytosis and Anthrax: Implications for Bacterial Sepsis?

**DOI:** 10.33696/immunology.3.090

**Published:** 2021

**Authors:** Joshua S. Mytych, Zijian Pan, A. Darise Farris

**Affiliations:** 1 Arthritis and Clinical Immunology Program, Oklahoma Medical Research Foundation (OMRF), 825 NE 13th St., Oklahoma City, OK 73104, USA; 2 Department of Microbiology and Immunology, University of Oklahoma Health Sciences Center (OUHSC), 940 Stanton L. Young Blvd, Oklahoma City, OK 73104, USA

**Keywords:** Efferocytosis, *Bacillus anthracis*, Sepsis, Apoptotic cell clearance, Edema toxin

## Introduction

*Bacillus anthracis* (Ba) is a gram-positive, rod-shaped, spore- and toxin-forming bacterium. While mainly an herbivore pathogen, human infection with Ba spores can occur through a number of routes including cutaneous, gastrointestinal, injectional, or inhalational [1]. The deadliest form of anthrax exposure is through inhalation of Ba spores, leading to systemic dissemination of the bacteria, with mortality ranging from 45% to 90% [2,3]. Current evidence suggests that sepsis, defined as a life-threatening organ dysfunction caused by a dysregulated host response to infection [4], is likely to play a critical role in death from systemic anthrax [5].

Significant increases in leukocyte apoptosis in the innate and adaptive lineages including natural killer, dendritic, T, and B cells, as well as monocytes and macrophages are known to accompany sepsis infections though the mechanisms leading to immune cell apoptosis are not well-characterized [6]. Although a primary role for Ba Lethal Toxin (LT) in killing human immune cells was long-suspected, several subsequent studies have failed to convincingly demonstrate LT’s capacity to kill most types of human leukocytes *in vitro* [7-11]. A recent review highlighted potential alternative mechanisms of elevated immune cell death in the setting of sepsis, including increases in cytokines, steroids, or host damage-associated molecular patterns (DAMPs), as well as changes in cell survival and death pathways for Fas signaling [12]. Regardless of the mechanisms responsible for immune cell death in late-stage anthrax and sepsis, the resulting cellular corpses must be efficiently cleared to prevent the propagation of inflammation. Release of normally sequestered host cell DAMPs can arise from improper clearance of apoptotic cells as a consequence of the progression of apoptosis to later stages of cell death. Secondary necrosis is a late-stage process whereby the cell membrane permeabilizes, leading to the release of intracellular contents including cytosolic and nuclear proteins (e.g. HMGB-1, nucleosomes, etc.) that promote inflammation after their recognition by various pattern recognition receptors [13,14]. Thus, a growing interest has developed around the mechanisms, consequences and interventions of clearing apoptotic cells during sepsis and systemic anthrax infection.

In our recent publication, we found that Ba Edema Toxin (ET) inhibits the proper clearance of apoptotic neutrophils by human macrophages. Ba’s virulence factors include its outer poly-D glutamic acid capsule, pathogen-associated molecular patterns (PAMPs) including peptidoglycan, lipoteichoic acid and nucleic acids, as well as two A-B type toxins: LT and ET. ET is composed of Protective Antigen (PA) and Edema Factor (EF), and acts to increase cyclic adenosine monophosphate (cAMP) to supra-physiologic levels, while LT is a metalloprotease composed of PA + Lethal Factor (LF) and acts to degrade MAPK signaling intermediates [15]. PA is the cell-binding component of both ET and LT that is required for entry of the toxins into host cells, as well as translocation of the toxins from the endosome into the cytosol of target cells. The toxins act to stunt the initial immune response, allowing bacteria to reach high numbers [16]. ET modulates responding macrophages by suppressing phagocytosis [17], chemotaxis [18], and as our recent publication showed, the clearance of apoptotic cells. How bacterial components affect the clearance of apoptotic cells during sepsis or systemic anthrax infection is currently not well characterized.

## Clearance of Apoptotic Cells – Efferocytosis

Efferocytosis is the process by which phagocytes recognize, ingest, and respond to apoptotic cells. This process typically maintains tissue homeostasis; however, defects in efferocytosis are linked to autoimmune disease [19], tissue injury [20], and impaired resolution of inflammation [21]. Efferocytosis is a multi-stage process that begins with the initial recognition of apoptotic cells, which occurs through either binding of macrophage receptors to the apoptotic membrane (direct recognition) or through soluble mediators that bridge macrophage receptors to the apoptotic membrane (indirect recognition). After initial binding, apoptotic cells are ingested by a coordinated, Rac1-dependent process that clears the cellular debris, and following processing, macrophages undergo phenotypic changes at the transcriptional level to modulate macrophage response (reviewed in [22]). Physiologic clearance of sudden increases in apoptotic cells, relevant to sepsis, is thought to be mediated by a combination of lymphoid organ and tissue-resident macrophages [23,24]. Efferocytic macrophages in humans exhibit high expression of CD163, CD206, MerTK, and other pro-efferocytic markers [25]. *In vitro* these cells are often modeled by polarizing macrophages through exposure to glucocorticoids (e.g. dexamethasone) or a combination of cytokines (e.g. IL-4 and IL-13, or IL-10) [26]. Interestingly, glucocorticoids have been an historic treatment modality for septic patients [27]. Perhaps part of the mechanism of action involves the promotion of efferocytosis and/or functional modulation of macrophage activation state. There is evidence that clearance of apoptotic cells results in functional modulation of the macrophage phenotype to an alternative/anti-inflammatory state as part of a pro-resolving mechanism following inflammation [26]. Although defects in efferocytosis are known to contribute to a variety of host inflammatory diseases, the role of defective efferocytosis in sepsis and/or systemic anthrax infection is less characterized.

## Edema Toxin Inhibits Efferocytosis

Given the important role of efferocytosis in regulating host inflammation, our article asked whether ET, a factor known to contribute to systemic pathology during inhalational anthrax, alters the capacity of macrophages to perform efferocytosis [28]. To summarize these findings, primary human macrophages treated with dexamethasone developed a pro-efferocytic M2 phenotype, noted by increased expression of MerTK, CD163, CD206, and αVβ5. We found that macrophages exposed to ET *in vitro* at physiologically-relevant concentrations had a reduced capacity to clear apoptotic neutrophils. Further, we found that ET induced changes to the cytoskeletal machinery responsible for engulfing apoptotic cells, including focal adhesion kinase (FAK), Calmodulin-dependent protein kinases K1, K4 (CamK1, K4), vasodilator-stimulated phosphoprotein (VASP), and Rac1, which act to regulate actin organization for cell engulfment. Understanding the mechanisms whereby ET, and possibly other bacterial factors, contribute to defective efferocytosis could pave the way for new treatment strategies in late-stage anthrax and other septic infections. Intriguingly, another bacterial factor, Ba peptidoglycan, induces many key clinical markers of systemic host pathology, including vascular occlusion, organ damage, and disseminated intravascular coagulation (DIC) in baboon [29] and rat [30] models of anthrax, suggesting that ET may not be the only bacterial factor affecting efferocytosis during infection.

## Defects in Efferocytosis are Linked to Sepsis

**Figure 1:**
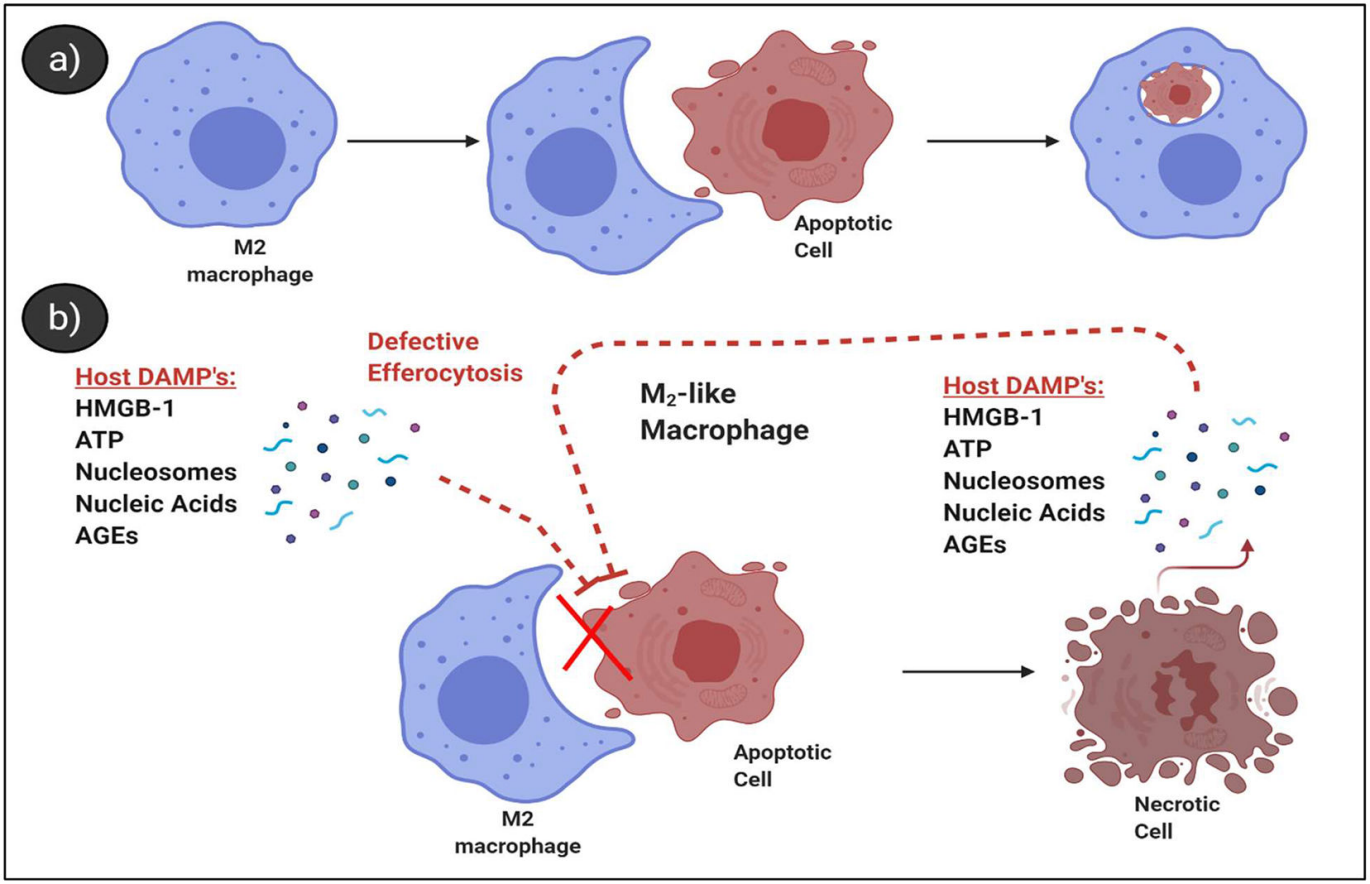
Proposed host-driven mechanism leading to defective efferocytosis. **a)** Under normal conditions apoptotic cells are efficiently cleared by macrophages that recognize, ingest, and process them **b)** Schematic representation of host DAMPs and proposed feed-back loop on efferocytosis from secondary necrotic cells. Uncleared apoptotic cells release various mediators (e.g. nuclear and cytosolic factors) that reduce the capacity of macrophages to efferocytose subsequent apoptotic cells, prolonging inefficient clearance and potentiating a pro-inflammatory response. Currently unknown are the mechanism(s) that lead to defects in the clearance of apoptotic cells. Figure was created with Biorender.com.

Despite being a defined disease process for over 3,000 years, there is no approved biologic therapy for the treatment of sepsis, with over 150 clinical trials failing to-date, suggesting an incomplete understanding of the pathology behind the septic disease process [29,30]. Preventing immune cell apoptosis is a well-described treatment strategy for sepsis [31]; However, studies addressing apoptotic cell clearance during sepsis, or in inhalational anthrax are few [12,32,33]. The molecular mechanisms leading to an accumulation of apoptotic lymphocytes in sepsis or the role uncleared apoptotic cells may play in sepsis progression are unclear [34]. Under steady-state conditions, apoptotic cells are rarely detected, even in organs with high turn-over of cells such as the spleen, due to efficient clearance [35]. This is in contrast to sepsis, where apoptotic lymphocytes and other apoptotic cell populations are detectable or abundant in the spleen, lungs, kidneys, and other organs [36], indicating a reduction of their clearance [37]. Defects in the clearance of apoptotic cells permit their progression into secondary necrosis and release of intracellular pro-inflammatory DAMPs [38]. Interestingly, levels of circulating DAMPs are known to perpetuate host organ damage in sepsis [39,40] and are implicated in causing defective efferocytosis [22] (Figure 1). Nucleosomes are a host-derived DAMP thought to be released from uncleared apoptotic cells. Circulating levels of nucleosomes have been shown to correlate with key features of sepsis, including intra-alveolar hemorrhage and the presence of micro-thrombi in an *E. coli* baboon sepsis model [41]. In addition to host DAMPs, PAMPs such as Ba peptidoglycan have been shown to model similar features of sepsis [42] and modulate host immune cells [43,44]. Multiple animal models of sepsis have demonstrated that maintaining and/or increasing receptors (Stabilin-1 [45]) or soluble mediators (Gas6 [46], pro-efferocytic lipids [47], or thrombospondin [48]) that promote efferocytosis improves survival and reduces negative outcomes. Whether this is an effective strategy in inhalational anthrax remains to be investigated.

## Common Features of Anthrax- and Non-anthrax Mediated Sepsis

Sepsis is a leading cause of mortality worldwide, contributing approximately 5 million deaths globally each year with nearly half due to gram positive bacteria (~48%) [49]. Pathology of sepsis infection is complex, with often multiple contributors including bacterial PAMPs (LPS, LTA, PGN), and host-derived DAMPs (HMGB1, S100A proteins), and coagulopathies which culminate in multiple organ dysfunction syndrome and death [29,50]. Anthrax-mediated sepsis differs from other more common forms of sepsis in that its infection course is established. Briefly, inhaled spores get transported to the mediastinal lymph nodes, germinate, and rapidly disseminate in the blood stream to high numbers [51]. The major role of Ba’s virulence factors, the outer poly-D-glutamic acid capsule, LT and ET, are to inhibit the initial host immune response, allowing the bacteria to multiply unimpeded [52]. In animal models addressing the role of toxins, they appear to exert differential effects that are largely model-dependent. Toxin contribution to late-stage infection, particularly in context of anti-toxin administration remains unclear [53,54]. PAMPs and DAMPs are likely to contribute to defective efferocytosis in anthrax-mediated sepsis, however, the addition of ET, which further reduces apoptotic cell clearance, may contribute to its particularly severe and rapid infection. Ba is an obligate pathogen, with near 100% fatality if untreated in otherwise healthy individuals [55]. In contrast, other infectious agents such as *pseudomonas* are usually not pathogenic for healthy individuals and plague those who are hospitalized for other illnesses, with hospital-acquired sepsis accounting for nearly 50% of sepsis cases [49]. There are likely other PAMPs shared across organisms, as well as host DAMPs in addition to ET that contribute to defective efferocytosis. Regardless of the route or causative agent there appears to be underlying disease mechanisms in common for both anthrax- and non-anthrax mediated sepsis once the bacteria are fulminant.

## Efferocytosis May Help or Harm the Host during Infection

While defective efferocytosis may be a feature of severe sepsis, how its modulation may impact late-stage infection is unclear. Currently, the literature is mixed and context-dependent, with some studies indicating that efferocytosis may increase the risk of infection due to immune suppression (e.g. higher TGF-beta, IL-10) [56,57], while others show increased bacterial clearance [58,59]. Data from *in vitro* modeling of how tissue-resident macrophages respond to diverse stimuli including bacterial PAMPs and apoptotic cells are also inconsistent due to differences in the studied macrophage sub-types, stimuli, and experimental methodologies [26,60-62]. One treatment paradigm currently being developed (Allocetra) aims to polarize macrophages to an alternative/pro-efferocytic phenotype by allogeneic apoptotic cell therapy. In a mouse cecal ligation and puncture model, in which affected mice exhibit sepsis with multiple organ dysfunction syndromes, severe cytokine storm, and acute kidney injury, Allocetra in combination with the antibiotic ertapenem, reduced disease severity by 50%, and improved survival by 10-fold (6% vs 60%) [63].

## Conclusion and Perspectives

Large increases in apoptotic cells are a known feature common to sepsis and inhalational anthrax. Prior treatment strategies have been aimed primarily at combating infection, blocking excessive host inflammation, and preventing host cell apoptosis. Emerging studies suggest that harnessing the efferocytic and pro-resolving activities of endogenous macrophages may be another tool in the arsenal for treating sepsis and reducing mortality. We found that ET reduced efferocytosis, and altered cytoskeletal signaling in human macrophages. ET, combined with other bacterial factors and host DAMPs, act to inhibit efferocytosis and augment host inflammation. Prior work from other labs demonstrate bacterial components, namely LPS, or host-derived DAMPs can reduce efferocytic capacity in a potential feed-forward loop. However, whether other bacterial and/or host-derived factors affect this process and which inhibitory factors predominate during infection, are yet to be investigated. Work on multiple fronts suggest that modulating efferocytosis could reduce negative outcomes in sepsis, including reducing the systemic inflammatory response and host mortality in animal sepsis models. Based on current literature, sepsis from various bacterial origins appear to maintain common underlying disease mechanism(s), in part manifested as an elevation in inflammatory cytokines, defects in apoptotic cell clearance, organ damage and high mortality. Thus, we hope to shed light on this topic to stimulate further research exploring efferocytosis in sepsis and systemic anthrax infection.
